# Jetset: selecting the optimal microarray probe set to represent a gene

**DOI:** 10.1186/1471-2105-12-474

**Published:** 2011-12-15

**Authors:** Qiyuan Li, Nicolai J Birkbak, Balazs Gyorffy, Zoltan Szallasi, Aron C Eklund

**Affiliations:** 1 Center for Biological Sequence Analysis, Technical University of Denmark, 2800 Lyngby, Denmark; 2 Children's Hospital Informatics Program at the Harvard-MIT Division of Health Sciences and Technology (CHIP@HST), Harvard Medical School, Boston, MA 02115, USA; 3 Joint Research Laboratory of the Hungarian Academy of Sciences and the Semmelweis University, Semmelweis University 1st Dept of Pediatrics, H-1083 Budapest, Hungary

## Abstract

**Background:**

Interpretation of gene expression microarrays requires a mapping from probe set to gene. On many Affymetrix gene expression microarrays, a given gene may be detected by multiple probe sets, which may deliver inconsistent or even contradictory measurements. Therefore, obtaining an unambiguous expression estimate of a pre-specified gene can be a nontrivial but essential task.

**Results:**

We developed scoring methods to assess each probe set for specificity, splice isoform coverage, and robustness against transcript degradation. We used these scores to select a single representative probe set for each gene, thus creating a simple one-to-one mapping between gene and probe set. To test this method, we evaluated concordance between protein measurements and gene expression values, and between sets of genes whose expression is known to be correlated. For both test cases, we identified genes that were nominally detected by multiple probe sets, and we found that the probe set chosen by our method showed stronger concordance.

**Conclusions:**

This method provides a simple, unambiguous mapping to allow assessment of the expression levels of specific genes of interest.

## Background

Gene expression microarrays are designed to measure the relative abundance of gene transcripts by detecting sequence-specific hybridization between a fixed DNA probe and a labeled RNA target. For intentional or unintentional reasons, some probes may detect multiple genes, and some genes may be detected by multiple probes. For many types of analysis, this is not a problem. For example, microarrays can be used as a screening tool to identify differentially expressed genes associated with a biological phenotype. In this case, a probe set with an expression pattern of interest can be mapped to a particular gene or set of transcripts using annotations from the manufacturer or from others [1-4].

However, some analyses require expression estimates for a specific set of genes, where the abundance of specific splice isoforms is unimportant. For example, in studies of breast tumors it may be important to determine the expression level of the genes ESR1 and ERBB2, which correspond to the clinically important estrogen receptor (ER) and Her2 proteins [5]. On the HG-U133A platform, there are nine probe sets designed to detect ESR1, but only one of these probe sets is strongly correlated with ER status determined by immunohistochemical methods [6]. As another example, one may wish to assess the expression of a set of genes in a previously defined signature or module; each gene may correspond to multiple Affymetrix probe sets.

At least three systematic approaches could be used to estimate a single expression value for a particular gene: 1) Use the average expression value of all probe sets that map to the gene. 2) Redefine the mapping between individual probes and probe sets, such that there is a one-to-one mapping between genes and redefined probe sets [4,7-11]. 3) From the set of probe sets that map to a particular gene, select a single, most representative probe set. The disadvantage of the first approach is that the signal from accurate probe sets can be corrupted by noise from inaccurate probe sets. The disadvantages of the second approach are a lack of a clear standard for defining the probe sets, unstable probe set definitions that may change as genome and transcriptome data are updated, and a requirement for raw data that may not always be available. Thus, the second approach adds a layer of complexity that may be acceptable when analyzing a single data set but complicates the comparison with results generated at different times or with different remappings. However, the third approach is conceptually simple, is likely to be more accurate than the first approach, uses only the stable, manufacturer-supplied identifiers, and can be readily applied to expression data for which the original probe-level data is unavailable. Therefore, we set out to produce a one-to-one mapping from each gene to its single, optimal probe set.

To evaluate the suitability of a probe set, we considered three factors. First, the probes in the probe set should respond specifically to the target gene and not to other genes. Several studies have explored the specificity of Affymetrix microarray probes and found that probe sets are most effective when their individual probes match their intended target [12]. Furthermore, probes that partially match other, unintended targets may deliver misleading results [13,14].

Second, the probe set should detect as many splice isoforms of the target gene as possible. The present work is primarily concerned with analytical problems in which the gene, but not the specific isoform, is specified. For this purpose the *overall *expression level of a gene, counting all functional splice isoforms, is the desired measurement. If the expression level of individual splice isoforms is desired, other tools are available [15].

Third, the probe set should query the target gene at a position near the 3' end of the corresponding transcripts. The microarrays considered in this work are designed to be used with an Eberwine-type target generation protocol. Because the reverse transcription and in vitro transcription steps are initiated at the poly-A tail, there is a 3' bias in the amount of labeled target that is generated [16]. Thus, probes that are too far from the 3' end of the target are likely to have a lower signal intensity, and for this reason most (but not all) probes are designed by Affymetrix to query their target within 600 bases of the 3' end of the transcript, or within 300 bases for the X3P array. In addition to a weaker signal, probes far from the 3' end of the gene are susceptible to false signal changes resulting from variations in RNA integrity [17].

We developed a method to score each probe set according to the three criteria described above. For genes that are detected by more than one probe set, we selected the highest-scoring probe set to represent that gene. We evaluated this one-to-one mapping by comparing measured gene expression levels to protein levels.

## Results

### Algorithm

We acquired probe sequences for four widely used human gene expression microarrays from Affymetrix: U95Av2, U133A, U133 Plus 2.0, and X3P. We used NCBI BLASTN to search the 25-base probe sequences for matches to the Refseq human cDNA database [18]. The BLASTN search was run with the default parameters, except that the word size was set to 8 to increase sensitivity. We used the maximum alignment score (bit score) between each probe and cDNA as an indication of hybridization affinity. We defined three levels of alignment: a *strong *alignment has a score between 48 and 51, indicating that at least 24 bases are identical and that the probe is very likely to detect the target. A *moderate *alignment has a score between 32 and 47, corresponding to an uninterrupted alignment of length 16 to 23 bases; the probe may or may not respond to the target. A *weak *alignment has a score less than 32 and is unlikely to respond to the target.

#### Specificity

A probe was considered to specifically detect a given gene if it strongly aligned to at least one transcript of the gene, but did not have a strong or moderate alignment to a transcript from any other gene. The gene specifically detected by the largest number of probes in a probe set is considered the *targeted gene *of the probe set. The *specificity score S_s _*of a probe set is the fraction of its probes that specifically detect the targeted gene.

#### Coverage

A transcript of the targeted gene was considered detected by a probe set if the transcript has a strong alignment to the majority of the probes in the probe set. The *coverage score S*_*c *_of a probe set is the fraction of all transcripts belonging to the targeted gene that are detected by the probe set.

#### Robustness

The *processivity requirement *for a probe-transcript alignment is the number of bases between the 5' end of the alignment and the 3' end of the transcript sequence; this corresponds to the length of labeled target that must be synthesized by in vitro transcription to reach the query region. The overall processivity requirement *N *of a probe set is the median processivity requirement for all strong alignments between probes in the probe set and transcripts in the targeted gene. We define the *robustness score S*_*r *_of a probe set as the probability that synthesis of the target up to the processivity requirement is achieved without interruption:

$$S_{r}={\left(1-p\right)}^{N}$$

Here, *p *is the probability of the IVT synthesis being interrupted at each base, due to either transcript degradation or lack of enzyme processivity. The value of *p *is likely to be variable in clinical specimens, but for simplicity we use a value corresponding to the manufacturer's design criteria: 1/300 for the X3P array, or 1/600 for the other arrays.

#### Overall score

We define the overall score *S_o _*as the product of the three scores described above:

$$S_{o}=S_{s}S_{c}S_{r}$$

For a given gene, the probe set targeting this gene with the highest overall score is selected to represent the gene.

### Testing

#### Calculation of jetset scores

**Figure 1:**
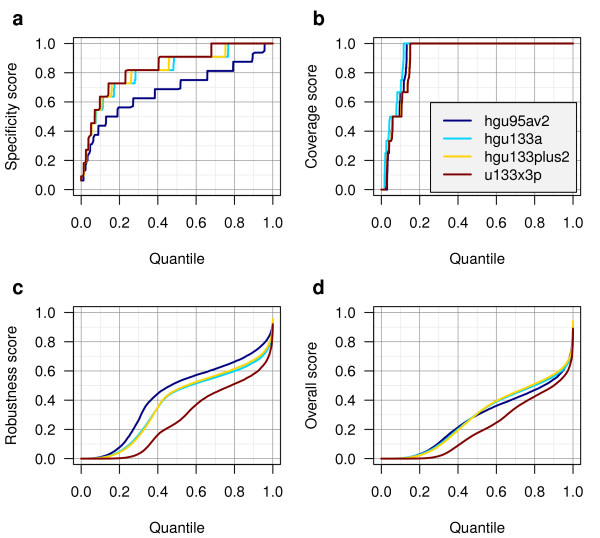
**Distribution of probe set scores**. Probe set score distributions are displayed for four Affymetrix human gene expression microarray platforms: a) Specificity score, b) Coverage score, c) Robustness score, and d) Overall score. Each platform features a different number of probe sets; therefore the X-axis is plotted as a quantile to enable comparison.

We calculated scores corresponding to four Affymetrix human gene expression microarray platforms that are highly represented in the GEO database. In general, we observed higher specificity scores for the newer (U133) platforms than for the older HG-U95Av2 platform, likely reflecting the more accurate genome data available when the newer arrays were designed (Figure 1a). The coverage scores were similar on each platform, with ~85% of probe sets achieving a perfect coverage score (Figure 1b). The robustness scores were notably lower in the X3P platform, perhaps reflecting the difficulty of meeting the design criteria for this platform by placing the probe set within 300 bases of the 3' end of the transcript (Figure 1c). Because of this, the overall score distribution was also lower in the X3P platform (Figure 1d).

#### Comparison with ER and HER2 status in breast tumors

**Figure 2:**
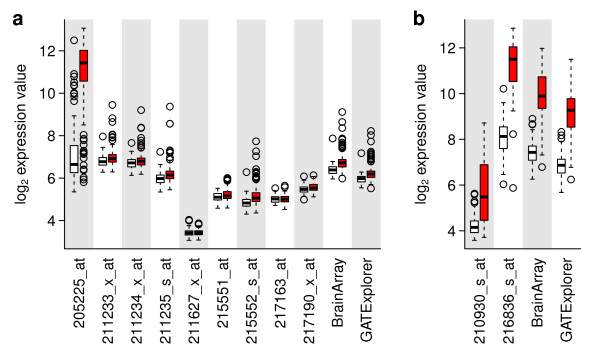
**Comparison of gene expression with histological measurements**. Publicly-available breast cancer data sets with histological annotation were used to compare probe set accuracy. a) ESR1 expression levels were estimated using each of the nine manufacturer-defined probe sets (Table 1), or by custom probe sets defined by BrainArray or GATExplorer. For each probe set, a pair of box plots indicates the distribution of expression levels in 77 ER-negative tumors (white) and in 209 ER-positive tumors (red) by protein quantification. b) In a separate data set, ERBB2 expression levels were estimated similarly, and compared in 99 HER2-negative (white) tumors and 33 HER2-positive (red) breast tumors.

We evaluated the Jetset mappings using a publicly-available data set representing 286 breast tumor specimens with HG-U133A microarray measurements and ER protein status as determined by ligand-binding assay, enzyme immunoassay, or immunohistochemistry [19]. In this data, we expected that the ER protein status should correlate with the ESR1 gene expression level. Using annotations from the manufacturer, from Bioconductor, and from the analysis described here, we identified nine probe sets that could possibly detect the ESR1 gene (Table 1). Of these, the Jetset algorithm identified 205225_at as the best probe set to detect the ESR1 gene. We observed that 205225_at was the only ESR1 probe set to show strongly differential expression correlated with protein levels (Figure 2a).

**Table 1:** Probe sets that query the ESR1 and ERBB2 genes

	Gene annotation		Jetset scores			
	
Probe set	Affy/Bioc	Jetset	*S_s_*	*S_c_*	*S_r_*	*S_o_*
**205225_at**	ESR1	ESR1	0.91	1.00	0.64	**0.58**
211233_x_at	ESR1	ESR1	0.64	1.00	0.00	0.00
211234_x_at	ESR1	ESR1	0.55	1.00	0.00	0.00
211235_s_at	ESR1	ESR1	0.91	1.00	0.00	0.00
211627_x_at	ESR1	--	--	--	--	--
215551_at	ESR1	--	--	--	--	--
215552_s_at	ESR1	ESR1	0.91	1.00	0.00	0.00
217163_at	ESR1	ESR1	0.27	0.00	0.00	0.00
217190_x_at	ESR1	ESR1	0.55	1.00	0.00	0.00
210930_s_at	ERBB2	ERBB2	0.82	1.00	0.00	0.00
**216836_s_at**	ERBB2	ERBB2	0.91	1.00	0.52	**0.48**

Gene annotations are from the manufacturer (Affy) or from Bioconductor (Bioc) or derived in the present analysis (Jetset). Probe sets that did not meet the Jetset annotation specificity criteria are indicated by "--". Each probe set was scored for specificity (*S_s_*), coverage (*S_c_*), robustness against mRNA degradation (*S_r_*), and a combined overall score (*S_o_*). For each gene, the probe set with the highest overall score (in bold) is selected to represent that gene.

We also evaluated the performance of two alternative probe set definitions: the Brainarray "hgu133ahsentrezgcdf" and the GATExplorer "genemapperhgu133acdf", both of which redefine probe sets such that each queries an individual gene [4,8]. In both cases, the remapped probe set querying ESR1 failed to detect strong differential expression between ER-positive and ER-negative tumors (Figure 2a).

We next evaluated a second publicly-available breast cancer data set for which clinical HER2 status based on protein immunohistochemistry or on fluorescence in situ hybridization (FISH) was annotated in 132 breast tumors [20]. Here we expected the HER2 status to correlate with the ERBB2 gene, which encodes the HER2 protein. The manufacturer, Bioconductor, and Jetset annotations agree that ERBB2 is queried by two probe sets (Table 1). We evaluated these two probe sets as well as two remapped ERBB2 probe sets as described above, and found that the probe set selected by the Jetset algorithm, 216836_s_at, best distinguished between HER2-positive and HER2-negative tumors (Figure 2b).

#### Concordance of a gene expression module in breast tumors

**Figure 3:**
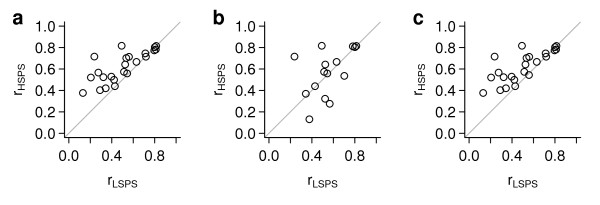
**Coherence of a gene expression module**. The genes in the previously-described CIN70 signature are consistently correlated in multiple data sets and measurement platforms. For the 23 CIN70 genes that are queried by more than one probe set, we identified the highest-scoring probe set (HSPS) and the lowest-scoring probe set (LSPS), and calculated the Pearson correlation coefficient (r) between each of these probe sets and the median of all CIN70 genes. The HSPS and LSPS were identified by either a) overall score, b) sensitivity score, or c) robustness score.

We previously identified a set of 70 co-expressed genes whose expression is associated with chromosomal instability (CIN) in cancer [21]. These "CIN70" genes were identified based on their correlation in multiple microarray data sets using various platforms; therefore we expect the genes in this module to be generally correlated with each other in other cancer data sets. Furthermore, for any of the CIN70 genes, we expect that the probe set selected by the Jetset algorithm should be more likely to be correlated with the other genes in the signature than a probe set with a lower score. For each of the 286 tumors in the first breast cancer data set, we defined the CIN70 score as the median expression value of all 94 probe sets that query the CIN70 genes. For the 23 CIN70 genes that were queried by more than one probe set, we calculated the Pearson correlation between individual probe set expression values and the CIN70 score. We compared these correlation values between the highest-scoring probe set and the lowest-scoring probe set, and found that the highest-scoring probe sets (the ones selected by the Jetset algorithm) were generally better correlated (Figure 3a).

We also performed a similar analysis using the individual components of the overall score: specificity, coverage, and robustness. The specificity score alone did not perform well in identifying highly-correlated probe sets (Figure 3b). The coverage score alone did not vary enough to perform meaningful analysis (data not shown). However, the robustness score alone was able to essentially reproduce the performance of the overall score (Figure 3c).

#### Implementation

The individual scores calculated by these methods can be found on the project website [22]. Additionally, we developed an R package called "jetset" that contains these scores along with functions to retrieve the highest-scoring probe set for a given gene. The R package is available from our website and has been submitted to Bioconductor [23]. The Jetset data and package will be updated following the Bioconductor release cycle (~ every six months).

## Discussion

We have developed a heuristic method for rating probe sets and a tool for choosing a single representative probe set for a given gene. The intended use of this tool is to rapidly select probe sets to assess the expression of a previously-defined set of genes in a microarray data set. We do not necessarily expect that every probe set in our mapping is the optimal choice, but our results suggest that our mapping performs reasonably well at least in our test cases.

Our approach is intended to complement, not replace, probe-level remapping methods. We and others have previously used probe-level remapping to redefine probe sets such that each reflect a single gene, with generally acceptable results. However, when performing analysis across a large number of data sets, we found probe-level remapping to be inconvenient because we were unable to include many data sets for which we did not have the raw probe-level data.

Our evaluation of ESR1 and ERBB2 probe sets indicates that Jetset chooses the probe set that best matches protein or FISH-based histological results, confirming the utility of our method. Furthermore, the identification of these specific probe sets is in agreement with previous results [6]. Suprisingly to us, the probe-level remapping approaches performed substantially worse for the ESR1 gene, likely because this gene is queried by an uncharacteristically large number of probe sets rendered ineffective by their distance from the 3' end of the transcript. We also evaluated our method by analyzing the coherence of genes in the previously described CIN70 module and again found that the probe sets selected by Jetset outperformed the probe sets with lower scores. Interestingly, in both sets of evaluations, it appears to be the robustness score that primarily drives the probe set selection, likely because a large fraction of probe sets have robustness scores that are very low.

Jetset is designed as a general tool, and thus it is not necessarily optimized for specific projects. The specificity score and coverage score are calculated without consideration of the relative abundance of splice isoforms. This abundance varies from one tissue type to another, and a sophisticated user may wish to consider this aspect when selecting a probe set. Also, by providing the three individual scores, we have left the possibility for the user to decide on an overall scoring scheme most suitable for a specific project. For example, the robustness score might be more important for use with samples that are known to be highly degraded, e.g. paraffin-embedded tumor specimens. Furthermore, this approach is not valid for data from all types of microarray. For example, whole-transcript amplification methodologies, as used in the newer arrays from Affymetrix, do not require that the probe be located near the 3' end of the transcript.

It might be possible to use this method or similar to identify probe sets as good or bad, regardless of the mapped gene. However, sample quality can vary greatly depending on the type of specimen, and hybridization specificity can vary due to choice of protocol; as discussed above both of these factors can affect the relative importance of the scores. Therefore, deriving an absolute score cutoff to separate good probe sets from bad probe sets would not be generally applicable to all experiments, and we did not attempt to do this.

## Conclusions

We have described a method to calculate principled, unbiased quality scores for Affymetrix probe sets, and to use these scores to define a simple, unambiguous mapping from gene to probe set.

## Methods

All analysis was performed using the R statistical environment and is recorded in Additional File 1.

## Authors' contributions

QL developed software and performed analysis. NJB performed analysis. BG and ZS participated in study design. ACE developed software, performed analysis, and drafted the manuscript. All authors read and approved the final manuscript.

## Supplementary Material

Additional file 1 **Sweave document**. This document contains the R code used to generate the results and figures in the paper. Click here for file

## References

[B1] Durinck S Spellman PT Birney E Huber W Mapping identifiers for the integration of genomic datasets with the R/Bioconductor package biomaRt Nat Protoc 2009 4 8 1184 1191 10.1038/nprot.2009.97 PMC3159387 19617889

[B2] Leong HS Yates T Wilson C Miller CJ ADAPT: a database of affymetrix probesets and transcripts Bioinformatics 2005 21 10 2552 2553 10.1093/bioinformatics/bti359 15746287

[B3] Liu G Loraine AE Shigeta R Cline M Cheng J Valmeekam V Sun S Kulp D Siani-Rose MA NetAffx: Affymetrix probesets and annotations Nucleic Acids Res 2003 31 1 82 86 10.1093/nar/gkg121 PMC165568 12519953

[B4] Risueno A Fontanillo C Dinger ME De Las Rivas J GATExplorer: genomic and transcriptomic explorer; mapping expression probes to gene loci, transcripts, exons and ncRNAs BMC Bioinformatics 2010 11 221 10.1186/1471-2105-11-221 PMC2875241 20429936

[B5] Wirapati P Sotiriou C Kunkel S Farmer P Pradervand S Haibe-Kains B Desmedt C Ignatiadis M Sengstag T Schutz F Meta-analysis of gene expression profiles in breast cancer: toward a unified understanding of breast cancer subtyping and prognosis signatures Breast Cancer Res 2008 10 4 R65 10.1186/bcr2124 PMC2575538 18662380

[B6] Gong Y Yan K Lin F Anderson K Sotiriou C Andre F Holmes FA Valero V Booser D Pippen JE Jr Determination of oestrogen-receptor status and ERBB2 status of breast carcinoma: a gene-expression profiling study Lancet Oncol 2007 8 3 203 211 10.1016/S1470-2045(07)70042-6 17329190

[B7] Carter SL Eklund AC Mecham BH Kohane IS Szallasi Z Redefinition of Affymetrix probe sets by sequence overlap with cDNA microarray probes reduces cross-platform inconsistencies in cancer-associated gene expression measurements BMC Bioinformatics 2005 6 107 10.1186/1471-2105-6-107 PMC1127107 15850491

[B8] Dai M Wang P Boyd AD Kostov G Athey B Jones EG Bunney WE Myers RM Speed TP Akil H Evolving gene/transcript definitions significantly alter the interpretation of GeneChip data Nucleic Acids Res 2005 33 20 e175 10.1093/nar/gni179 PMC1283542 16284200

[B9] Gautier L Moller M Friis-Hansen L Knudsen S Alternative mapping of probes to genes for Affymetrix chips BMC Bioinformatics 2004 5 111 10.1186/1471-2105-5-111 PMC514699 15310390

[B10] Liu H Zeeberg BR Qu G Koru AG Ferrucci A Kahn A Ryan MC Nuhanovic A Munson PJ Reinhold WC AffyProbeMiner: a web resource for computing or retrieving accurately redefined Affymetrix probe sets Bioinformatics 2007 23 18 2385 2390 10.1093/bioinformatics/btm360 17660211

[B11] Lu J Lee JC Salit ML Cam MC Transcript-based redefinition of grouped oligonucleotide probe sets using AceView: high-resolution annotation for microarrays BMC Bioinformatics 2007 8 108 10.1186/1471-2105-8-108 PMC1853115 17394657

[B12] Mecham BH Klus GT Strovel J Augustus M Byrne D Bozso P Wetmore DZ Mariani TJ Kohane IS Szallasi Z Sequence-matched probes produce increased cross-platform consistency and more reproducible biological results in microarray-based gene expression measurements Nucleic Acids Res 2004 32 9 e74 10.1093/nar/gnh071 PMC419626 15161944

[B13] Eklund AC Friis P Wernersson R Szallasi Z Optimization of the BLASTN substitution matrix for prediction of non-specific DNA microarray hybridization Nucleic Acids Res 2010 38 4 e27 10.1093/nar/gkp1116 PMC2831327 19969549

[B14] Okoniewski MJ Miller CJ Hybridization interactions between probesets in short oligo microarrays lead to spurious correlations BMC Bioinformatics 2006 7 276 10.1186/1471-2105-7-276 PMC1513401 16749918

[B15] Moll AG Lindenmeyer MT Kretzler M Nelson PJ Zimmer R Cohen CD Transcript-specific expression profiles derived from sequence-based analysis of standard microarrays PLoS One 2009 4 3 e4702 10.1371/journal.pone.0004702 PMC2650090 19277110

[B16] Auer H Lyianarachchi S Newsom D Klisovic MI Marcucci G Kornacker K Chipping away at the chip bias: RNA degradation in microarray analysis Nat Genet 2003 35 4 292 293 10.1038/ng1203-292 14647279

[B17] Eklund AC Szallasi Z Correction of technical bias in clinical microarray data improves concordance with known biological information Genome Biol 2008 9 2 R26 10.1186/gb-2008-9-2-r26 PMC2374720 18248669

[B18] Pruitt KD Tatusova T Maglott DR NCBI Reference Sequence (RefSeq): a curated non-redundant sequence database of genomes, transcripts and proteins Nucleic Acids Res 2005 33 Database D501 504 10.1093/nar/gki025 PMC539979 15608248

[B19] Wang Y Klijn JG Zhang Y Sieuwerts AM Look MP Yang F Talantov D Timmermans M Meijer-van Gelder ME Yu J Gene-expression profiles to predict distant metastasis of lymph-node-negative primary breast cancer Lancet 2005 365 9460 671 679 10.1016/S0140-6736(05)17947-1 15721472

[B20] Hess KR Anderson K Symmans WF Valero V Ibrahim N Mejia JA Booser D Theriault RL Buzdar AU Dempsey PJ Pharmacogenomic predictor of sensitivity to preoperative chemotherapy with paclitaxel and fluorouracil, doxorubicin, and cyclophosphamide in breast cancer J Clin Oncol 2006 24 26 4236 4244 10.1200/JCO.2006.05.6861 16896004

[B21] Carter SL Eklund AC Kohane IS Harris LN Szallasi Z A signature of chromosomal instability inferred from gene expression profiles predicts clinical outcome in multiple human cancers Nat Genet 2006 38 9 1043 1048 10.1038/ng1861 16921376

[B22] jetset http://www.cbs.dtu.dk/biotools/jetset/

[B23] Gentleman RC Carey VJ Bates DM Bolstad B Dettling M Dudoit S Ellis B Gautier L Ge Y Gentry J Bioconductor: open software development for computational biology and bioinformatics Genome Biol 2004 5 10 R80 10.1186/gb-2004-5-10-r80 PMC545600 15461798

