# Supplementary material for: Jetset: selecting the optimal microarray probe set to represent a gene
Source: BMC Bioinformatics. 2011 Dec 15;12:474. doi: 10.1186/1471-2105-12-474 (PMC3266307; doi:10.1186/1471-2105-12-474)
Supplement: Additional file 1 — Sweave document. This document contains the R code used to generate the results and figures in the paper. [file 1471-2105-12-474-S1.PDF]

Jetset: selecting the optimal microarray probe set to represent a gene: Supplementary information

Qiyuan Li, Nicolai J Birkbak, Balazs Gyorffy, Zoltan Szallasi, and Aron C Eklund

November 23, 2011

1 Load and prepare packages and data

1.1 Load necessary packages

```
> library(affy)
> library(hgu133ahsentrezgcdf)
> library(genemapperhgu133acdf)
> library(hgu133a.db)
> library(jetset)
```

1.2 Load previously prepared data

Wang et al breast cancer x 286

```
> #load('~/sbge_cancer/data/batch/wang.batch.RData')
> load('~/sbge_cancer/data/exprs/wang.rma.RData')
```

Hess et al breast cancer x 133

```
> #load('~/sbge_cancer/data/batch/hess.batch.RData')
> load('~/sbge_cancer/data/exprs/hess.rma.RData')
```

Probe set annotations provided by Affymetrix

```
> (load('~/sbge_cancer/info/annotation/ann.u133a.na29.RData'))

[1] "ann.u133a"

> str(ann.u133a)

'data.frame':        22283 obs. of  3 variables:
 $ symbol: chr  "DDR1" "RFC2" "HSPA6" "PAX8" ...
 $ gene  : chr  "discoidin domain receptor tyrosine kinase 1" "replication factor C (activator 1)
 $ chr   : chr  "chr6p21.3" "chr7q11.23" "chr1q23" "chr2q12-q14" ...
```

2 Define some plotting functions

These are taken from the non-public "aronmisc" package. The first function is used in all figures; the remaining functions are used only in Figure 1.

```
> label.panel <- function (txt, xoff = 1, yoff = xoff, cex = 8/6, font = 2)
+ {
+     x <- grconvertX(0, from = "nfc") + (xoff * strwidth("M"))
+     y <- grconvertY(1, from = "nfc") - (yoff * strheight("M"))
+     text(x, y, labels = txt, font = font, xpd = TRUE, cex = cex,
+         adj = c(0, 1))
+ }
```

The following functions are used for Figure 1

```
> fdce.list <-  function (x, n = 100, plot = TRUE, na.last = NA, norm = FALSE,
+     xlab = ifelse(norm, "Quantile", "Index"), ylab = deparse(substitute(x)),
+     ...)
+ {
+     aa <- lapply(x, fdce, plot = FALSE, n = n, na.last = na.last,
+         norm = norm)
+     if (plot) {
+         listplot(aa, xlab = xlab, ylab = ylab, ...)
+     }
+     invisible(aa)
+ }
```

```
> fdce <- function (x, n = 100, plot = TRUE, na.last = NA, norm = FALSE,
+     xlab = ifelse(norm, "Quantile", "Index"), ylab = deparse(substitute(x)),
+     ...)
+ {
+     a <- approx2(sort(x, na.last = na.last), n = n)
+     if (norm) {
+         a$x <- a$x/max(a$x)
+     }
+     if (plot) {
+         plot(a, xlab = xlab, ylab = ylab, ...)
+     }
+     invisible(a)
+ }
```

```
> ## approx2 eliminates redundant points from fdce plots
> approx2 <- function (x, y = NULL, n = 100)
+ {
+     x <- xy.coords(x, y)
+     ll <- nrow(x)
+     dx <- diff(x$x)/(max(x$x) - min(x$x))
+     dy <- diff(x$y)/(max(x$y) - min(x$y))
+     d <- c(0, cumsum(sqrt((dx^2) + (dy^2))))
+     tgt <- seq(0, d[length(d)], length = n)
+     wh <- unique(sapply(tgt, function(a) which.min(abs(d - a))))
+     x$x <- x$x[wh]
+     x$y <- x$y[wh]
+     x
+ }
```

```
> listplot <- function (x, col = 1:6, pch = 1, lwd = 1, lty = 1, type = "p",
+     xlab = "x", ylab = "y", add = FALSE, ...)
+ {
+     x <- lapply(x, xy.coords)
+     col <- rep(col, length.out = length(x))
+     pch <- rep(pch, length.out = length(x))
+     lwd <- rep(lwd, length.out = length(x))
+     lty <- rep(lty, length.out = length(x))
+     type <- rep(type, length.out = length(x))
+     if (!add) {
+         xlim <- range(sapply(x, function(y) y$x))
+         ylim <- range(sapply(x, function(y) y$y))
+         plot(xlim, ylim, type = "n", xlab = xlab, ylab = ylab,
+             ...)
+     }
+     for (i in seq(along = x)) {
+         lines(x[[i]]$x, x[[i]]$y, col = col[i], lty = lty[i],
+             lwd = lwd[i], pch = pch[i], type = type[i])
+     }
+ }
```

```
> grid2 <- function (nx = 2, ny = 2, col = c("gray50", "gray90"), lty = 1,
+     lwd = 1)
+ {
+     col = rep(col, length.out = 2)
+     lty = rep(lty, length.out = 2)
+     lwd = rep(lwd, length.out = 2)
+     atx <- axTicks(1)
+     dx <- atx[2] - atx[1]
+     atx <- seq(atx[1] - dx, atx[length(atx)] + dx, by = dx/nx)
+     atx <- atx[atx > par("usr")[1]]
+     atx <- atx[atx < par("usr")[2]]
+     atx <- atx[!(atx %in% axTicks(1))]
+     abline(v = atx, col = col[2], lty = lty[2], lwd = lwd[2])
+     aty <- axTicks(2)
+     dy <- aty[2] - aty[1]
+     aty <- seq(aty[1] - dy, aty[length(aty)] + dy, by = dy/ny)
+     aty <- aty[aty > par("usr")[3]]
+     aty <- aty[aty < par("usr")[4]]
+     aty <- aty[!(aty %in% axTicks(2))]
+     abline(h = aty, col = col[2], lty = lty[2], lwd = lwd[2])
+     grid(col = col[1], lty = lty[1], lwd = lwd[1])
+ }
```

3 Results for each platform

```
> scorelist <- list(
+   hgu95av2 = jscores('hgu95av2'),
+   hgu133a = jscores('hgu133a'),
+   hgu133plus2 = jscores('hgu133plus2'),
+   u133x3p = jscores('u133x3p')
+ )
```

```
> platformStats <- data.frame(
+   nProbesets = sapply(scorelist, nrow),
+   nScored = sapply(scorelist, function(x) sum(!is.na(x$EntrezID))),
+   fractionScored = sapply(scorelist, function(x) mean(!is.na(x$EntrezID))),
+   uniqueGenes = sapply(scorelist, function(x) length(na.omit(unique(x$EntrezID))))
+ )
> platformStats
```

```
            nProbesets nScored fractionScored uniqueGenes
hgu95av2         12625   10644      0.8430891        8422
hgu133a          22283   17977      0.8067585       12147
hgu133plus2      54675   32180      0.5885688       18844
u133x3p          61359   34574      0.5634707       18754
```

Median scores for each platform

```
> platformMedianScores <- data.frame(
+   specificity = sapply(scorelist, function(x) median(x$specificity, na.rm = TRUE)),
+   coverage = sapply(scorelist, function(x) median(x$coverage, na.rm = TRUE)),
+   robust = sapply(scorelist, function(x) median(x$robust, na.rm = TRUE)),
+   overall = sapply(scorelist, function(x) median(x$overall, na.rm = TRUE))
+ )
> platformMedianScores
```

```
            specificity coverage    robust   overall
hgu95av2      0.6875000        1 0.5226338 0.2979845
hgu133a       0.9090909        1 0.4658134 0.3080022
hgu133plus2   0.9090909        1 0.4720711 0.3063104
u133x3p       0.9090909        1 0.2484977 0.1749521
```

Median scores for each platform, optimal probe sets only

```
> scorelist.opt <- lapply(scorelist, function(x) x[!duplicated(x$EntrezID) & !is.na(x$EntrezID),
> platformMedianScoresOpt <- data.frame(
+   specificity = sapply(scorelist.opt, function(x) median(x$specificity, na.rm = TRUE)),
+   coverage = sapply(scorelist.opt, function(x) median(x$coverage, na.rm = TRUE)),
+   robust = sapply(scorelist.opt, function(x) median(x$robust, na.rm = TRUE)),
+   overall = sapply(scorelist.opt, function(x) median(x$overall, na.rm = TRUE))
+ )
> platformMedianScoresOpt
```

```
            specificity coverage    robust   overall
hgu95av2      0.7500000        1 0.5591610 0.3541588
hgu133a       0.9090909        1 0.5252557 0.4194074
hgu133plus2   0.9090909        1 0.5568340 0.4555915
u133x3p       0.9090909        1 0.4442566 0.3511507
```

4 Figure 1: Distribution of scores within each platform

Scores listed by platform (slbp)

```
> slbp.specificity <- lapply(scorelist, function(x) x$specificity)
> slbp.coverage <- lapply(scorelist, function(x) x$coverage)
> slbp.robust <- lapply(scorelist, function(x) x$robust)
> slbp.overall <- lapply(scorelist, function(x) x$overall)
```

Plotting function

```
> myCol <- c("#00007F", "#00D4FF", "#FFD400", "#7F0000")
> p01 <- function(x, ylim = 0:1, ...) {
+   fdce.list(x,
+     norm = TRUE, type = 'l', ylim = ylim, col = myCol, lwd = 2,
+     panel.first = grid2(lwd = 0.5), ...)
+ }
```

```
> par(mfrow = c(2, 2), cex = 1, las = 1,
+   mar = c(4, 4, 2, 1) + 0.1)
> p01(slbp.specificity,
+   ylab = 'Specificity score')
> label.panel('a')
> p01(slbp.coverage,
+   ylab = 'Coverage score')
> label.panel('b')
> legend('bottomright', legend = names(scorelist),
+   lty = 1, col = myCol, lwd = 2, bg = 'gray95', inset = 0.02)
> p01(slbp.robust,
+   ylab = 'Robustness score')
> label.panel('c')
> p01(slbp.overall,
+   ylab = 'Overall score')
> label.panel('d')
```

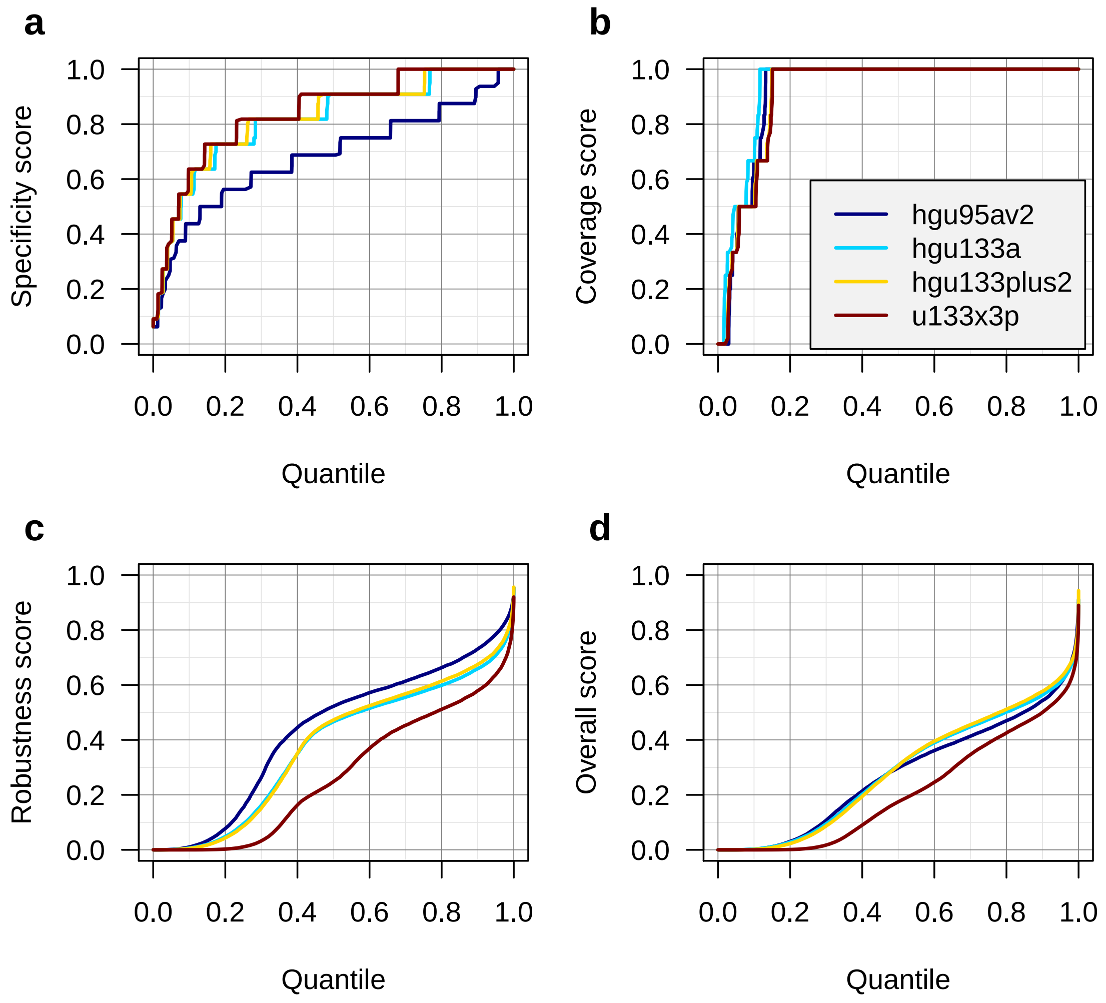

a
b
c
d
Specificity score
Coverage score
Robustness score
Overall score
Quantile
hgu95av2
hgu133a
hgu133plus2
u133x3p
0.0
0.2
0.4
0.6
0.8
1.0

5 Calculate Brainarray and GATExplorer expression values

BrainArray

```
> #wang.batch@cdfName <- "hgu133ahsentrezgcdf"
> #wang.ba.rma <- rma(wang.batch)
> #hess.batch@cdfName <- "hgu133ahsentrezgcdf"
> #hess.ba.rma <- rma(hess.batch)
> #save(wang.ba.rma, file = 'wang.ba.rma.RData')
> #save(hess.ba.rma, file = 'hess.ba.rma.RData')
> load('wang.ba.rma.RData')
> load('hess.ba.rma.RData')
```

GATExplorer

```
> #wang.batch@cdfName <- "genemapperhgu133acdf"
> #wang.ge.rma <- rma(wang.batch)
> #hess.batch@cdfName <- "genemapperhgu133acdf"
> #hess.ge.rma <- rma(hess.batch)
> #save(wang.ge.rma, file = 'wang.ge.rma.RData')
> #save(hess.ge.rma, file = 'hess.ge.rma.RData')
> load('wang.ge.rma.RData')
> load('hess.ge.rma.RData')
```

```
> dim(exprs(wang.rma))
```

```
[1] 22283   286
```

```
> dim(exprs(wang.ba.rma))
```

```
[1] 12146   286
```

```
> dim(exprs(wang.ge.rma))
```

```
[1] 12576   286
```

Confirm that samples are in the same order

```
> all(sampleNames(wang.rma) == sampleNames(wang.ge.rma))
```

```
[1] TRUE
```

```
> all(sampleNames(wang.rma) == sampleNames(wang.ba.rma))
```

```
[1] TRUE
```

6 Table 1: Jetset scores for ESR1 and ERBB2 probe sets

6.1 ESR1

According to Jetset, these probe sets query ESR1

```
> esr1.probesets.jetset <- rownames(jscores('hgu133a', symbol = 'ESR1'))
> esr1.probesets.jetset
```

```
[1] "205225_at"   "211235_s_at" "211233_x_at" "211234_x_at" "215552_s_at"
[6] "217190_x_at" "217163_at"
```

According to Affymetrix annotation:

```
> esr1.probesets.affy <- rownames(ann.u133a)[which(ann.u133a$symbol == 'ESR1')]
> esr1.probesets.affy
```

```
[1] "205225_at"   "211233_x_at" "211234_x_at" "211235_s_at" "211627_x_at"
[6] "215551_at"   "215552_s_at" "217163_at"   "217190_x_at"
```

According to Bioconductor:

```
> esr1.probesets.bioc <- names(which(unlist(as.list(hgu133aSYMBOL)) == 'ESR1'))
> esr1.probesets.bioc
```

```
[1] "205225_at"   "211233_x_at" "211234_x_at" "211235_s_at" "211627_x_at"
[6] "215551_at"   "215552_s_at" "217163_at"   "217190_x_at"
```

Affymetrix and Bioconductor agree:

```
> setequal(esr1.probesets.affy, esr1.probesets.bioc)
```

```
[1] TRUE
```

Jetset has two fewer:

```
> setdiff(esr1.probesets.affy, esr1.probesets.jetset)
```

```
[1] "211627_x_at" "215551_at"
```

```
> jscores('hgu133a', probeset = esr1.probesets.affy)
```

```
            nProbes EntrezID process specificity coverage       robust
205225_at        11     2099   266.0   0.9090909        1 6.416558e-01
211235_s_at      11     2099  4565.0   0.9090909        1 4.931581e-04
211233_x_at      11     2099  4630.0   0.6363636        1 4.424848e-04
211234_x_at      11     2099  4630.0   0.5454545        1 4.424848e-04
215552_s_at      11     2099  5148.0   0.9090909        1 1.864853e-04
217190_x_at      11     2099  4880.5   0.5454545        1 2.913595e-04
217163_at        11     2099  6273.0   0.2727273        0 2.855375e-05
211627_x_at      11     <NA>      NA          NA       NA           NA
215551_at        11     <NA>      NA          NA       NA           NA
                 overall symbol
205225_at   0.5833234938   ESR1
211235_s_at 0.0004483256   ESR1
211233_x_at 0.0002815813   ESR1
211234_x_at 0.0002413554   ESR1
215552_s_at 0.0001695321   ESR1
217190_x_at 0.0001589234   ESR1
217163_at   0.0000000000   ESR1
211627_x_at           NA   <NA>
215551_at             NA   <NA>
```

6.2 ERBB2

According to Jetset

```
> erbb2.probesets.jetset <- rownames(jscores('hgu133a', symbol = 'ERBB2'))
> erbb2.probesets.jetset
```

```
[1] "216836_s_at" "210930_s_at"
```

According to Affymetrix annotation:

```
> erbb2.probesets.affy <- rownames(ann.u133a)[which(ann.u133a$symbol == 'ERBB2')]
> erbb2.probesets.affy
```

```
[1] "210930_s_at" "216836_s_at"
```

According to Bioconductor:

```
> erbb2.probesets.bioc <- names(which(unlist(as.list(hgu133aSYMBOL)) == 'ERBB2'))
> erbb2.probesets.bioc
```

```
[1] "210930_s_at" "216836_s_at"
```

Nice that we all agree here.

```
> jscores('hgu133a', probeset = erbb2.probesets.affy)
```

```
            nProbes EntrezID process specificity coverage      robust
216836_s_at      11     2064     387   0.9090909        1 0.524380298
210930_s_at      11     2064    3486   0.8181818        1 0.002982937
                overall symbol
216836_s_at 0.476709362  ERBB2
210930_s_at 0.002440584  ERBB2
```

7 Figure 2: ESR1 and ERBB2 probe sets vs. histological measurements

7.1 Get expression values using BrainArray (Entrez gene) and GATExplorer (GeneMapper) remapped probe sets

ESR1

```
> wang.ba.esr1 <- exprs(wang.ba.rma)["2099_at", ]
> wang.ge.esr1 <- exprs(wang.ge.rma)["ENSG00000091831", ]
```

ERBB2

```
> hess.ba.erbb2 <- exprs(hess.ba.rma)["2064_at", ]
> hess.ge.erbb2 <- exprs(hess.ge.rma)["ENSG00000141736", ]
```

Reality check; do BrainArray and GATExplorer agree?

```
> par(mfrow = c(1,2), cex = 1, mar = c(4,4,2,2)+0.1)
> plot(wang.ba.esr1, wang.ge.esr1,
+   xlab = 'BrainArray ESR1',
+   ylab = 'GATExplorer ESR1')
> plot(hess.ba.erbb2, hess.ge.erbb2,
+   xlab = 'BrainArray ERBB2',
+   ylab = 'GATExplorer ERBB2')
```

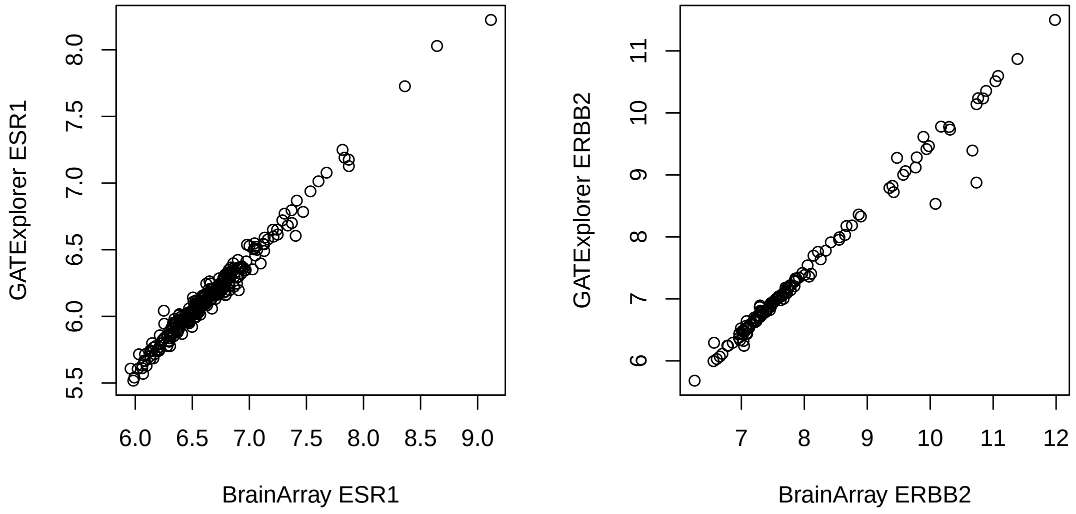

7.2 Histological calls

```
> ER.status <- wang.rma$ER
> levels(ER.status) <- c('N', 'P')
> table(ER.status)

ER.status
  N   P
 77 209

> HER2.status <- hess.rma$HERposTxt
> levels(HER2.status) <- c('N', 'P')
> table(HER2.status)

HER2.status
 N  P
99 33
```

7.3 Combine data for plotting

```
> esr1.matrix <- rbind(exprs(wang.rma)[esr1.probesets.affy, ],
+   BrainArray = wang.ba.esr1,
+   GATExplorer = wang.ge.esr1
+ )

> esr1.list <- list()
> for (i in 1:11) {
+   esr1.list[[(2 * i) - 1]] <- esr1.matrix[i, ][ER.status == 'N']
+   esr1.list[[(2 * i) - 0]] <- esr1.matrix[i, ][ER.status == 'P']
+ }

> erbb2.matrix <- rbind(exprs(hess.rma)[erbb2.probesets.affy, ],
+   BrainArray = hess.ba.erbb2,
+   GATExplorer = hess.ge.erbb2
+ )

> erbb2.list <- list()
> for (i in 1:4) {
+   erbb2.list[[(2 * i) - 1]] <- erbb2.matrix[i, ][which(HER2.status == 'N')]
+   erbb2.list[[(2 * i) - 0]] <- erbb2.matrix[i, ][which(HER2.status == 'P')]
+ }
```

7.4 Generate the figure

```
> layout( matrix(1:2, nrow = 1), widths = 2:1)
> par(las = 1, bty = 'l', mar = c(6, 3.5, 1, 1)+0.1,
+     mgp = c(1.8,0.5,0), tcl = -0.3)
> plot(c(0,33), range(esr1.list),
+   type = 'n', xaxs = 'i', axes = FALSE,
+   panel.first = rect(seq(0, 30, by = 6), 2, seq(3, 33, by = 6), 14,
+     col = 'gray90', border = NA),
+   xlab = '',
+   ylab = expression(paste(log[2], " expression value"))
+ )
> boxplot(esr1.list, col = c('white', 'red'), add = TRUE, axes = FALSE,
+   at = (1:33)[-seq(3, by = 3, length = 11)])
> axis(2)
> box()
> axis(1, tick = FALSE, las = 3,
+   at = seq(1.5, by = 3, length = 11),
+   labels = rownames(esr1.matrix))
> label.panel('a')
> plot(c(0,12), range(erbb2.list),
+   type = 'n', xaxs = 'i', axes = FALSE,
+   panel.first = rect(seq(0, 30, by = 6), 2, seq(3, 33, by = 6), 14,
+     col = 'gray90', border = NA),
+   xlab = '',
+   ylab = expression(paste(log[2], " expression value"))
+ )
> boxplot(erbb2.list, col = c('white', 'red'), add = TRUE, axes = FALSE,
+   at = (1:12)[-seq(3, by = 3, length = 11)])
> axis(2)
> box()
> axis(1, tick = FALSE, las = 3,
+   at = seq(1.5, by = 3, length = 4),
+   labels = rownames(erbb2.matrix))
> label.panel('b')
```

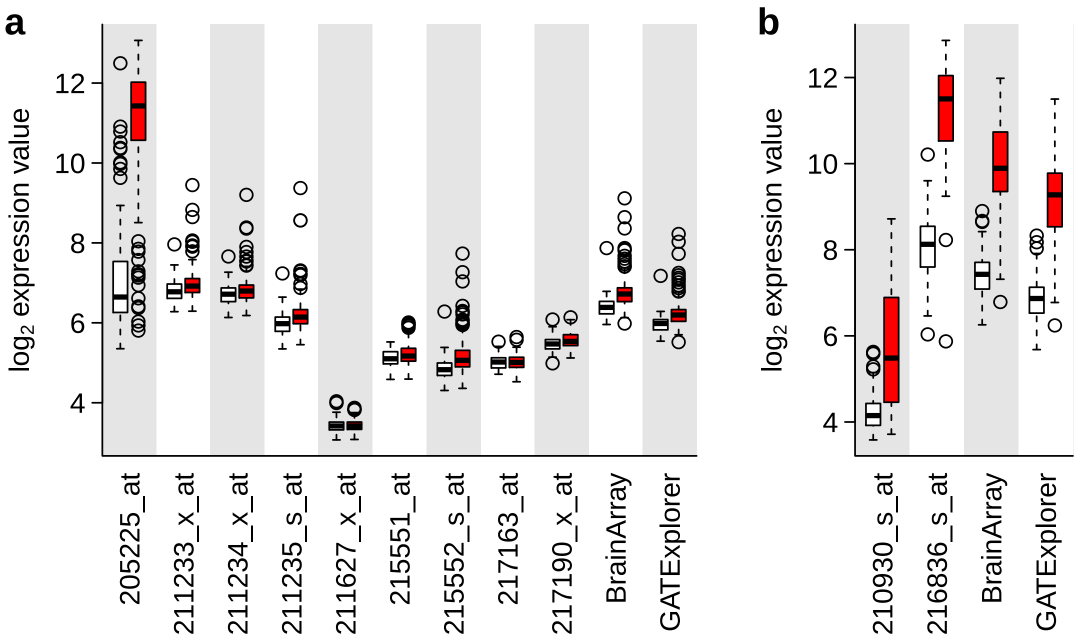

a
b
log2 expression value
12
10
8
6
4
205225_at
211233_x_at
211234_x_at
211235_s_at
211627_x_at
215551_at
215552_s_at
217163_at
217190_x_at
BrainArray
GATExplorer
210930_s_at
216836_s_at
BrainArray
GATExplorer

7.5 T tests

ESR1 vs. ER status

```
> data.frame(P = apply(esr1.matrix, 1, function(x) t.test(x ~ ER.status)$p.value))

                       P
205225_at   3.766543e-39
211233_x_at 3.049519e-05
211234_x_at 5.867152e-04
211235_s_at 1.055745e-06
211627_x_at 6.810385e-01
215551_at   4.034734e-04
215552_s_at 3.906265e-13
217163_at   8.065122e-01
217190_x_at 1.077518e-04
BrainArray  3.942716e-16
GATExplorer 1.585149e-13
```

ERBB2 vs. HER2 status

```
> data.frame(P = apply(erbb2.matrix, 1, function(x) t.test(x ~ HER2.status)$p.value))

                       P
210930_s_at 6.172162e-07
216836_s_at 3.494707e-14
BrainArray  2.619727e-13
GATExplorer 5.706632e-13
```

8 Figure 3: Correlation of genes in the CIN70 signature

8.1 Define the CIN70 signature

These genes were taken from Carter et al (2006) Nat Genetics, Supplementary Table 1.

```
> # These genes were taken from Carter et al (2006) Nat Genetics, Supplementary Table 1.
> # Compared to the original list, several gene symbols have been updated:
> # CDC2 --> CDK1
> # C20orf24/TGIF2 --> C20orf24 and TGIF2
> # CNAP1 --> NCAPD2 (?)
> # CDC45L --> CDC45
> # ch-TOG --> CKAP5
> # BRRN1 --> NCAPH
> # MTB --> MT1JP
> # TOPK --> PBK
> # FLJ10036 --> ZWILCH
> # GPIandMGC13096 --> GPI
> # SFRS2 --> SRSF2
> # STK6 --> AURKA
> # KIAA0286 --> TMEM194A
> cin70 <- c("TPX2", "PRC1", "FOXM1", "CDK1", "C20orf24", "TGIF2", "MCM2",
+ "H2AFZ", "TOP2A", "PCNA", "UBE2C", "MELK", "TRIP13", "NCAPD2", "MCM7",
+ "RNASEH2A", "RAD51AP1", "KIF20A", "CDC45", "MAD2L1", "ESPL1", "CCNB2",
+ "FEN1", "TTK", "CCT5", "RFC4", "ATAD2", "CKAP5", "NUP205", "CDC20",
+ "CKS2", "RRM2", "ELAVL1", "CCNB1", "RRM1", "AURKB", "MSH6", "EZH2", "CTPS", "DKC1",
+ "OIP5", "CDCA8", "PTTG1", "CEP55", "H2AFX", "CMAS", "NCAPH", "MCM10", "LSM4",
+ "MT1JP", "ASF1B", "ZWINT", "PBK", "ZWILCH", "CDCA3", "ECT2",
+ "CDC6", "UNG", "MTCH2", "RAD21", "ACTL6A", "GPI", "SRSF2", "HDGF", "NXT1",
+ "NEK2", "DHCR7", "AURKA", "NDUFAB1", "TMEM194A", "KIF4A")
```

Get Jetset scores for all probe sets querying CIN70 genes

```
> cin70.jscores <- jscores('hgu133a', symbol = cin70)
```

Consensus CIN70 signature value: median of all 94 probe sets

```
> cin70med <- apply(exprs(wang.rma)[rownames(cin70.jscores), ], 2, median)
> str(cin70med)

 Named num [1:286] 7.2 7 7.58 7.11 6.68 ...
 - attr(*, "names")= chr [1:286] "100.CEL.gz" "103.CEL.gz" "104.CEL.gz" "105.CEL.gz" ...
```

The correlation between the CIN70 median and each individual CIN70 probe set

```
> cin70.cor <- cor(t(exprs(wang.rma)[rownames(cin70.jscores), ]), cin70med)[, 1]
> str(cin70.cor)

 Named num [1:94] 0.715 0.815 0.803 0.666 0.803 ...
 - attr(*, "names")= chr [1:94] "204767_s_at" "208079_s_at" "38158_at" "213911_s_at" ...
```

8.2 For each gene, identify probe sets with highest and lowest overall score

Group Jetset scores by gene

```
> cin70.jscores.byGene <- split(cin70.jscores, cin70.jscores$EntrezID)
```

Which genes are queried by more than one probe set?

```
> has.multiple.probesets <- sapply(cin70.jscores.byGene, nrow) > 1
> table(has.multiple.probesets)

has.multiple.probesets
FALSE  TRUE
   45    23

> sbg.m <- cin70.jscores.byGene[has.multiple.probesets]
```

Get CIN70 correlations for highest and lowest-scoring probe sets

```
> hilo.overall <- data.frame(
+   best.probeset = sapply(sbg.m, function(x) rownames(x)[which.max(x$overall)]),
+   worst.probeset = sapply(sbg.m, function(x) rownames(x)[which.min(x$overall)]),
+   stringsAsFactors = FALSE
+ )
> hilo.overall$best.ps.cor <- cin70.cor[hilo.overall$best.probeset]
> hilo.overall$worst.ps.cor <- cin70.cor[hilo.overall$worst.probeset]
> hilo.overall

      best.probeset worst.probeset best.ps.cor worst.ps.cor
1717    201791_s_at    201790_s_at   0.5286787    0.3966516
1736      201479_at    216212_s_at   0.7021105    0.5353067
1994      201726_at    201727_s_at   0.4393734    0.4295494
2237    204767_s_at    204768_s_at   0.7146744    0.7185379
23165     212247_at    222382_x_at   0.6417052    0.5251788
23306     212621_at      212619_at   0.5230117    0.3221076
25804   202737_s_at    202736_s_at   0.5201183    0.2052411
2956      202911_at    211450_s_at   0.5662778    0.2752738
3014    205436_s_at    212525_s_at   0.7156722    0.2378865
3015    213911_s_at      200853_at   0.6662450    0.6306793
3068    200896_x_at    216484_x_at   0.5580323    0.5437578
4176    208795_s_at    210983_s_at   0.7135473    0.5605613
4751      204641_at    211080_s_at   0.8167423    0.4924244
5885    200608_s_at    200607_s_at   0.4985765    0.4218662
60436   216262_s_at    218724_s_at   0.4023539    0.2906736
6240    201477_s_at    201476_s_at   0.3764808    0.1310698
6241      201890_at    209773_s_at   0.7737099    0.7968552
6427    200754_x_at    200753_x_at   0.4198962    0.3433744
6790    208079_s_at    204092_s_at   0.8147695    0.8141049
7153      201292_at    201291_s_at   0.7450680    0.7131628
9700       38158_at      204817_at   0.8034945    0.8012324
983       203213_at    203214_x_at   0.7816037    0.8105444
990       203967_at    203968_s_at   0.5731681    0.5140910
```

Draw the figure

```
> par(mfrow = c(1,2), cex = 1)
> par(mar = c(4, 4, 1, 1) + 0.1, bty = 'l',
+   las = 1, mgp = c(2.5,0.5,0), tcl = -0.3)
> plot(hilo.overall$worst.ps.cor, hilo.overall$best.ps.cor,
+   xlab = 'PCC, lowest-scoring probe set',
+   ylab = 'PCC, highest-scoring probe set',
+   xlim = c(0, 1), ylim = c(0, 1),
+   panel.first = abline(0,1, col = 'gray70'))
```

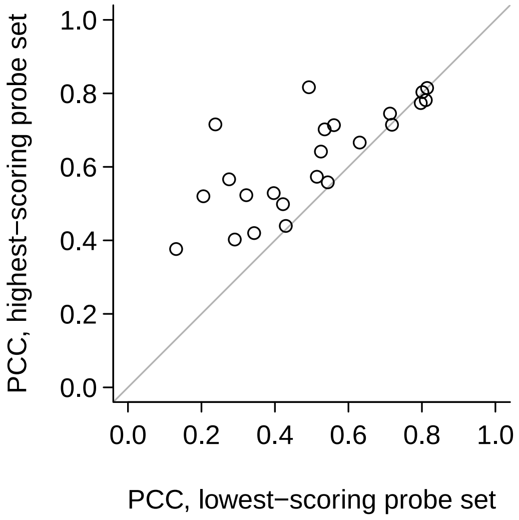

8.3 Would individual scores work just as well?

8.3.1 Specificity

Many probe sets have the same specificity score, so we consider only the genes with multiple probe sets with unique specificity scores.

```
> has.multiple.spec.scores <- sapply(sbg.m, function(x) length(unique(x$specificity)) > 1)
> table(has.multiple.spec.scores)

has.multiple.spec.scores
FALSE  TRUE
    8    15

> sbg.m2 <- sbg.m[has.multiple.spec.scores]
```

Get correlations for highest and lowest-scoring (specificity only) probe sets

```
> hilo.specificity <- data.frame(
+   best.probeset = sapply(sbg.m2, function(x) rownames(x)[which.max(x$specificity)]),
+   worst.probeset = sapply(sbg.m2, function(x) rownames(x)[which.min(x$specificity)]),
+   stringsAsFactors = FALSE
+ )
> hilo.specificity$best.ps.cor <- cin70.cor[hilo.specificity$best.probeset]
> hilo.specificity$worst.ps.cor <- cin70.cor[hilo.specificity$worst.probeset]
> hilo.specificity

      best.probeset worst.probeset best.ps.cor worst.ps.cor
1736    216212_s_at      201479_at   0.5353067    0.7021105
1994      201726_at    201727_s_at   0.4393734    0.4295494
23165     212247_at    222382_x_at   0.6417052    0.5251788
23306     212619_at      212621_at   0.3221076    0.5230117
2956    211450_s_at      202911_at   0.2752738    0.5662778
3014    205436_s_at    212525_s_at   0.7156722    0.2378865
3015    213911_s_at      200853_at   0.6662450    0.6306793
3068    200896_x_at    216484_x_at   0.5580323    0.5437578
4751      204641_at    211080_s_at   0.8167423    0.4924244
6240    201476_s_at    201477_s_at   0.1310698    0.3764808
6427    214882_s_at    200753_x_at   0.3687441    0.3433744
6790    208079_s_at    204092_s_at   0.8147695    0.8141049
9700       38158_at      204817_at   0.8034945    0.8012324
983     210559_s_at      203213_at   0.8114903    0.7816037
990       203967_at    203968_s_at   0.5731681    0.5140910
```

8.3.2 Coverage

Many probe sets have the same Coverage score, so we consider only the genes with multiple probe sets with unique Coverage scores.

```
> has.multiple.coverage.scores <- sapply(sbg.m, function(x) length(unique(x$coverage)) > 1)
> table(has.multiple.coverage.scores)
```

```
has.multiple.coverage.scores
FALSE  TRUE 
   21     2 
```

We cannot really draw conclusions from 2 data points.

8.3.3 Robustness

Get correlations for highest and lowest-scoring (robustness only) probe sets

```
> hilo.robust <- data.frame(
+   best.probeset = sapply(sbg.m, function(x) rownames(x)[which.max(x$robust)]),
+   worst.probeset = sapply(sbg.m, function(x) rownames(x)[which.min(x$robust)]),
+   stringsAsFactors = FALSE
+ )
> hilo.robust$best.ps.cor <- cin70.cor[hilo.robust$best.probeset]
> hilo.robust$worst.ps.cor <- cin70.cor[hilo.robust$worst.probeset]
> hilo.robust
```

```
      best.probeset worst.probeset best.ps.cor worst.ps.cor
1717    201791_s_at    201790_s_at   0.5286787    0.3966516
1736      201479_at    216212_s_at   0.7021105    0.5353067
1994      201726_at    201727_s_at   0.4393734    0.4295494
2237    204767_s_at    204768_s_at   0.7146744    0.7185379
23165     212247_at    222382_x_at   0.6417052    0.5251788
23306     212621_at      212619_at   0.5230117    0.3221076
25804   202737_s_at    202736_s_at   0.5201183    0.2052411
2956      202911_at    211450_s_at   0.5662778    0.2752738
3014    205436_s_at    212525_s_at   0.7156722    0.2378865
3015    213911_s_at      200853_at   0.6662450    0.6306793
3068    216484_x_at    200896_x_at   0.5437578    0.5580323
4176    208795_s_at    210983_s_at   0.7135473    0.5605613
4751      204641_at    211080_s_at   0.8167423    0.4924244
5885    200608_s_at    200607_s_at   0.4985765    0.4218662
60436   216262_s_at    218724_s_at   0.4023539    0.2906736
6240    201477_s_at    201476_s_at   0.3764808    0.1310698
6241      201890_at    209773_s_at   0.7737099    0.7968552
6427    200754_x_at    200753_x_at   0.4198962    0.3433744
6790    208079_s_at    204092_s_at   0.8147695    0.8141049
7153      201292_at    201291_s_at   0.7450680    0.7131628
9700       38158_at      204817_at   0.8034945    0.8012324
983       203213_at    203214_x_at   0.7816037    0.8105444
990       203967_at    203968_s_at   0.5731681    0.5140910
```

8.3.4 Combined figure

```
> par(mfrow = c(1,3), cex = 1)
> par(mar = c(3.5, 3.5, 1, 1) + 0.1, bty = 'l', pty = 's',
+   las = 1, mgp = c(2,0.5,0), tcl = -0.3)
> plot(hilo.overall$worst.ps.cor, hilo.overall$best.ps.cor,
+   xlab = expression(r[LSPS]),
+   ylab = expression(r[HSPS]),
+   xlim = c(0, 1), ylim = c(0, 1),
+   panel.first = abline(0,1, col = 'gray70'))
> label.panel('a')
> plot(hilo.specificity$worst.ps.cor, hilo.specificity$best.ps.cor,
+   xlab = expression(r[LSPS]),
+   ylab = expression(r[HSPS]),
+   xlim = c(0, 1), ylim = c(0, 1),
+   panel.first = abline(0,1, col = 'gray70'))
> label.panel('b')
> plot(hilo.robust$worst.ps.cor, hilo.robust$best.ps.cor,
+   xlab = expression(r[LSPS]),
+   ylab = expression(r[HSPS]),
+   xlim = c(0, 1), ylim = c(0, 1),
+   panel.first = abline(0,1, col = 'gray70'))
> label.panel('c')
```

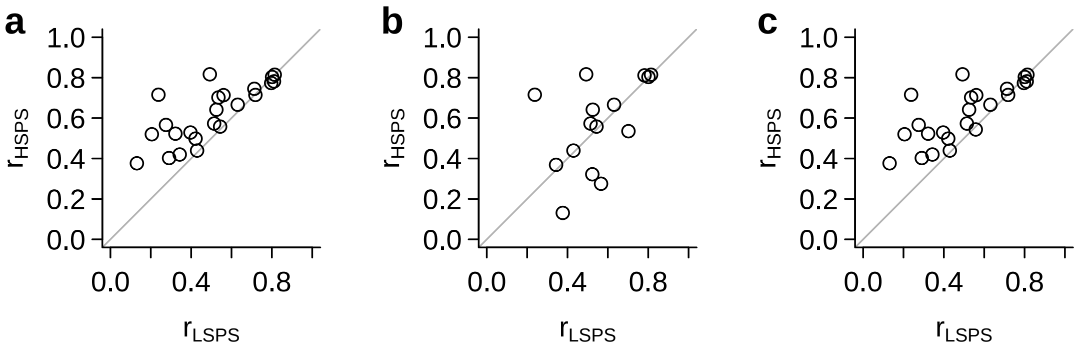

9 System information

9.1 R sessionInfo

The results in this file were generated using the following packages:

```
> sessionInfo()

R version 2.14.0 (2011-10-31)
Platform: x86_64-apple-darwin9.8.0/x86_64 (64-bit)

locale:
[1] en_US.UTF-8/en_US.UTF-8/en_US.UTF-8/C/en_US.UTF-8/en_US.UTF-8

attached base packages:
[1] stats     graphics  grDevices utils     datasets  methods   base

other attached packages:
 [1] jetset_0.99.2             hgu133a.db_2.6.3
 [3] org.Hs.eg.db_2.6.4        RSQLite_0.10.0
 [5] DBI_0.2-5                 AnnotationDbi_1.16.4
 [7] genemapperhgu133acdf_3.0   hgu133ahsentrezgcdf_14.1.0
 [9] affy_1.32.0               Biobase_2.14.0

loaded via a namespace (and not attached):
[1] affyio_1.22.0         BiocInstaller_1.2.1   IRanges_1.12.2
[4] preprocessCore_1.16.0 tools_2.14.0          zlibbioc_1.0.0
```

9.2 Other information

```
> system('uname -v', intern = TRUE)

[1] "Darwin Kernel Version 10.8.0: Tue Jun  7 16:32:41 PDT 2011; root:xnu-1504.15.3~1/RELEASE_X86

> system('pdftex --version', intern = TRUE)[1]

[1] "pdfTeX 3.1415926-1.40.11-2.2 (TeX Live 2010)"
```
